# Supplementary material for: Pleiotropy and epistasis within and between signaling pathways defines the genetic architecture of fungal virulence
Source: PLoS Genet. 2021 Jan 25;17(1):e1009313. doi: 10.1371/journal.pgen.1009313 (PMC7861560; doi:10.1371/journal.pgen.1009313)
Supplement: S1 Table — QTL-L and QTL-R refer to intergenic regions on chromosome 2 at 797,055—797,281 bp, between genes CNB02680 and CNB02690 and at 1,047,138—1,047,346 bp, between genes CNB03490 and CNB03500, respectively in the XL280a strain background [211]. (PDF) [file pgen.1009313.s001.pdf]

S1 Table. Genotypes of parental and transformant strains. QTL-L and QTL-R refer to intergenic regions on chromosome 2 at 797,055 – 797,281 bp, between genes *CNB02680* and *CNB02690* and at 1,047,138 – 1,047,346 bp, between genes *CNB03490* and *CNB03500*, respectively.

Strain	Name	Genotype	Source
SSA837	431α	wild type	[92]
SSB830	XL280**a**	wild type	[211]
SSA853	XL280αSS	*MAT*α, *ura5*::*NAT*	[92, 141]
PMY2408	CF1705	*MAT*α, QTL-R::*NEO* (431α)	this study
PMY2420	CF1706	*MAT*α, QTL-R::*NEO* (431α)	this study
PMY2432	CF1707	*MAT*α, QTL-R::*NEO* (431α)	this study
PMY2444	CF1730	*MAT***a**, QTL-L::*NAT* (XL280**a**)	this study
PMY2552	1a-12	*MAT*α, *SSK1*::*NAT* (431α)	this study
PMY2553	4b-2	*MAT***a**, *SSK1*::*NAT* (XL280**a**)	this study
PMY2554	5a-8	*MAT***a**, *SSK1*::*NAT* (XL280**a**)	this study
PMY2555	5b-1	*MAT***a**, *SSK1*::*NAT* (XL280**a**)	this study
