# Supplementary material for: Pleiotropy and epistasis within and between signaling pathways defines the genetic architecture of fungal virulence
Source: PLoS Genet. 2021 Jan 25;17(1):e1009313. doi: 10.1371/journal.pgen.1009313 (PMC7861560; doi:10.1371/journal.pgen.1009313)
Supplement: S2 Table — (PDF) [file pgen.1009313.s002.pdf]

Name	Primer sequence 5' to 3'	Purpose	Citation
***SSK1* gene disruption**			
CRISPR- Cas9			
Ssk1 P1 1000	CCCAAAAGAGGGTGGATGCT	construction of deletion cassette	
Ssk1 P2 1000	CTGGCCGTCGTTTTACGCCCCATTGTAACACCTCCA	construction of deletion cassette	
Ssk1 P3 1000	GTCATAGCTGTTTCCTGTGATGATCGAAGCCCGACAA	construction of deletion cassette	
Ssk1 P4 1000	TTATCCATTCTGCCGGTCGC	construction of deletion cassette	
Ssk1 P5 1000	GGCCTCAATGGCGTAGACTT	construction of deletion cassette	
Ssk1 P6 1000	CTGTTCAGGAGCAGAGCCAA	construction of deletion cassette	
Ssk1 sgF Cd1	GTCCCGTATCCTCGAGCTCAGGTTTTAGAGCTAGAAATAGCAAGTT	construction of gRNA cassette	
Ssk1 sgR Cd1	CTGAGCTCGAGGATACGGGACAACAGTATACCCTGCCGGTG	construction of gRNA cassette	
U6 F	TTTGCATTAGAACTAAAAACAAAGCA	construction of gRNA cassette	Fan and Lin 2018
gRNA R	TAAAACAAAAAAGCACCGACTCGGTGCC	construction of gRNA cassette	Fan and Lin 2018
U6 FLF	GGCTCAAAGAGCAGATCAATG	construction of gRNA cassette	Fan and Lin 2018
sgRNA FR	CCTCTGACACATGCAGCTCC	construction of gRNA cassette	Fan and Lin 2018
GPD1-P-F	CATGCATCTAGGTCTAGAAACC	amplifying Cas9 from pXL1-Cas9-HygB	
GPD1-T-R	CCTCTTCACGTGGACGCTCC	amplifying Cas9 from pXL1-Cas9-HygB	
Confirmation of transformants			
SSK1 F3	CGTCGGACTTCCCATAGCAT	detecting presence of *SSK1*	
SSK1 R3	ATCCTTCGTTTTGTCGGCCT	detecting presence of *SSK1*	
Cas9 intF	CGACCTCCGTCTCATCTACC	detecting presence of Cas9	
Cas9 intR	TTGAGGAGGGTGAGGTCTTG	detecting presence of Cas9	
SSK1 A	TAGTTCGAGGAAACGCGGAG	confirmation of boundaries, length	
SSK1 B	TGTTACGGAGCGATGGTGAC	confirmation of boundaries, length	
SSK1 NAT B	CTGGCGGAGGATAGAAGCTG	confirmation of boundaries, length	
SSK1 C	TCGCTTGCTAGCAGACTACG	confirmation of boundaries, length	
SSK1 D	ACTTGGATTAGGCATTGCATGT	confirmation of boundaries, length	
SSK1 NAT C	TCGGGTCAATTGTCTCAGTCG	confirmation of boundaries, length	
Sequencing			
SSK1 1F	CCAAGCATCAGTCATGCACC	sequencing of transformants	
SSK1 1R	CGCGTTACTTTTCTTCGCGT	sequencing of transformants	
SSK1 2F	AATGGAAAGACTCGGCTGGC	sequencing of transformants	
SSK1 2R	GGAAGCATTTGAGCCCCACA	sequencing of transformants	
SSK1 2R *NAT*	ATGTAAGTCCGCTCCTTCCC	sequencing of transformants	
SSK1 3F *NAT*	AGAATTCGCCCTTAGGCTGC	sequencing of transformants	
SSK1 3R *NAT*	CTCTGTCCAACGCACATCCA	sequencing of transformants	
SSK1 4F *NAT*	GCAACAGCCCATCCTTGTTG	sequencing of transformants	
SSK1 4R *NAT*	AAGAGCTTGCTCTCCGTCAG	sequencing of transformants	
SSK1 5F *NAT*	TTGTGGACTGGATACCGCAC	sequencing of transformants	
SSK1 5R	TAGGAGAGCTGGTCGACTCC	sequencing of transformants	
SSK1 6F	ACATGCAATGCCTAATCCAAGT	sequencing of transformants	
SSK1 6R	CAACCCCAACGGAGGAGAAA	sequencing of transformants	
SSK1 7F	GAGTTTGTTCATGGCAGAGGC	sequencing of transformants	
SSK1 7R	AGCCATGGTTTCTTTCCAGTAA	sequencing of transformants	
**Chr2 QTL fine mapping**			
CRISPR- Cas9			
QTL-L-5F	TGACTTCCCCTGGTTCCTAT	QTL left border *NAT* cassette	
QTL-L-5R	ACTGGCCGTCGTTTTACACCCGATGATTTTCCGAC	QTL left border *NAT* cassette	
QTL-L-3F	GTCATAGCTGTTTCCTGAAAGGAAGACGACGGGAAGT	QTL left border *NAT* cassette	
QTL-L-3R	AAAGCCAAGGACGAGGTCA	QTL left border *NAT* cassette	
QTL-R-5F	TGAAAACCGAAAACCCTGAC	QTL right border *NEO* cassette	
QTL-R-5R	ACTGGCCGTCGTTTTACACGCCTTCGTCACTCAAAAC	QTL right border *NEO* cassette	
QTL-R-3F	GTCATAGCTGTTTCCTGAGCAGTTGGCGTTAGCAGTT	QTL right border *NEO* cassette	
QTL-R-3R	GGACCTCTTCGTATTTTCAAGGAC	QTL right border *NEO* cassette	
QTL-L-F	ATGAGAGGTGGAACCGAGAG	QTL left border genotyping	
QTL-L-R	TCAAGTCCAACAAGCAGTGG	QTL left border genotyping	
QTL-R-F	AACTGGGCTGGGTCCATACA	QTL right border genotyping	
QTL-R-R	GTTCGGGAGCGTTCGTTAGA	QTL right border genotyping	
NAT-Southern-F	CGATACGGCTTACCGTTACAG	*NAT* southern probe	
NAT-Southern-R	GAGCTTGCTCTCCGTCAGATG	*NAT* southern probe	
NEO-Southern-F	GAAGGGACTGGCTGCTATTG	*NEO* southern probe	
NEO-Southern-R	GAACTCGTCAAGAAGGCGATA	*NEO* southern probe	
