# Supplementary figures and images for: Pleiotropy and epistasis within and between signaling pathways defines the genetic architecture of fungal virulence
Source: PLoS Genet. 2021 Jan 25;17(1):e1009313. doi: 10.1371/journal.pgen.1009313 (PMC7861560; doi:10.1371/journal.pgen.1009313)

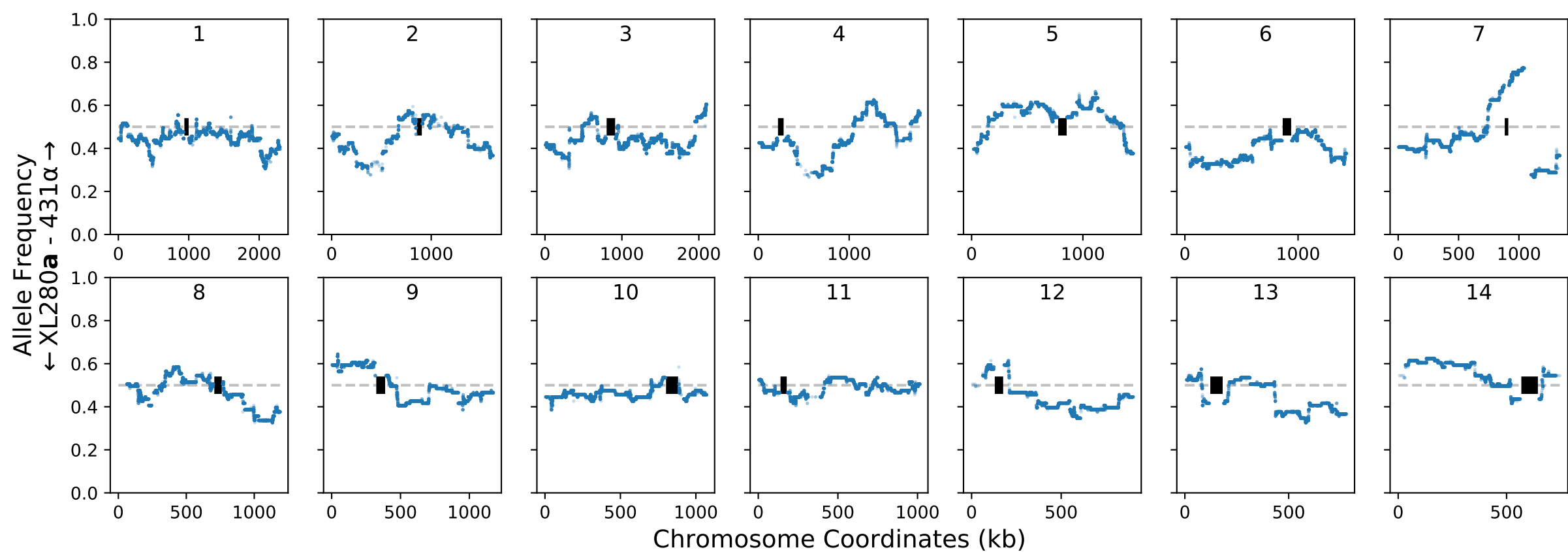

Supplement: S1 Fig — C. deneoformans strains XL280a and XL280αSS were crossed with 431α in a–α bisexual and α–α unisexual matings, generating 101 segregants. Between the parental strains there are 92,103 bi-allelic genetic variants (see Materials and methods) and these genetic variants are collapsed across the segregants, based on genetic exchange events, generating 3,108 unique haplotypes across the genome. The allele frequencies of these haplotypes (blue dots) per chromosome are shown for each of the 14 chromosomes (numbers denote chromosome). A horizontal, grey dashed line marks an allele frequency of 0.5. Centromere locations are marked by black rectangles. The bias present on the right of chromosome 7 is due to selectable genetic markers used to generate progeny from the α–α unisexual cross [141]. (PDF) [file pgen.1009313.s004.pdf]

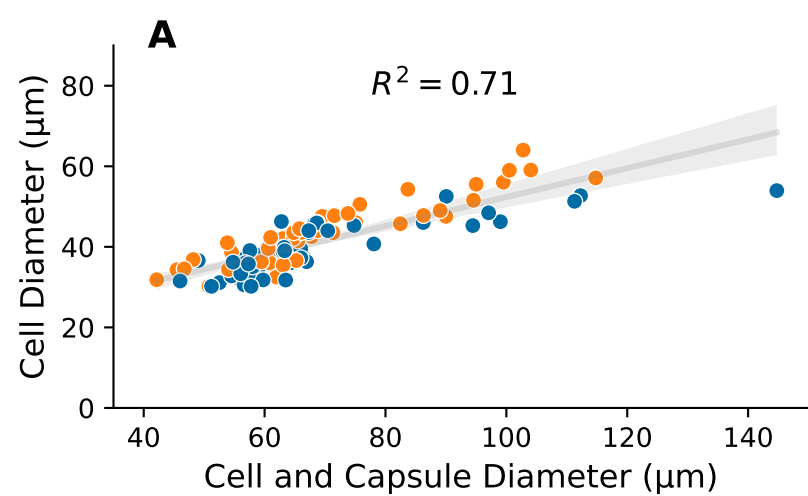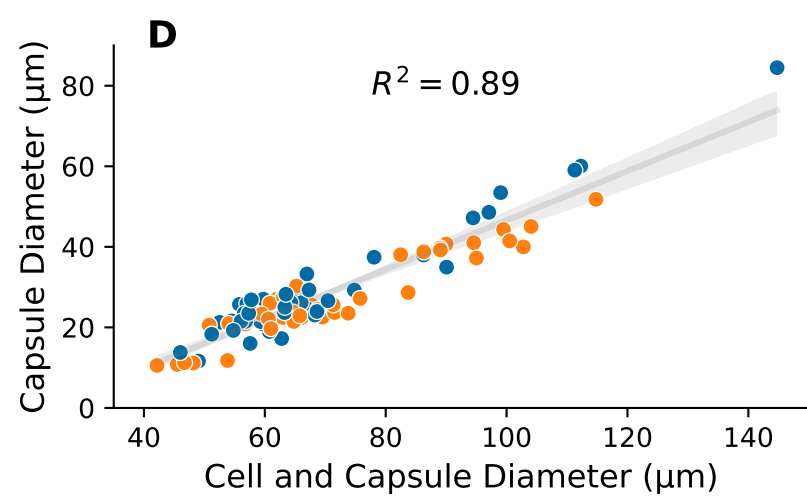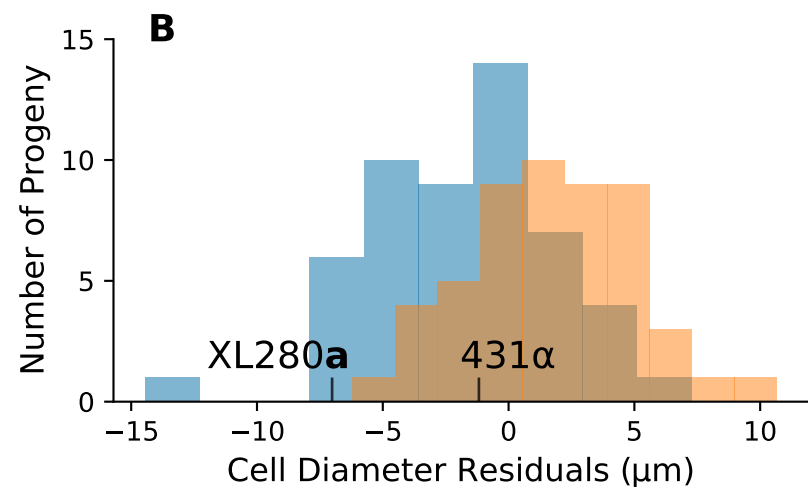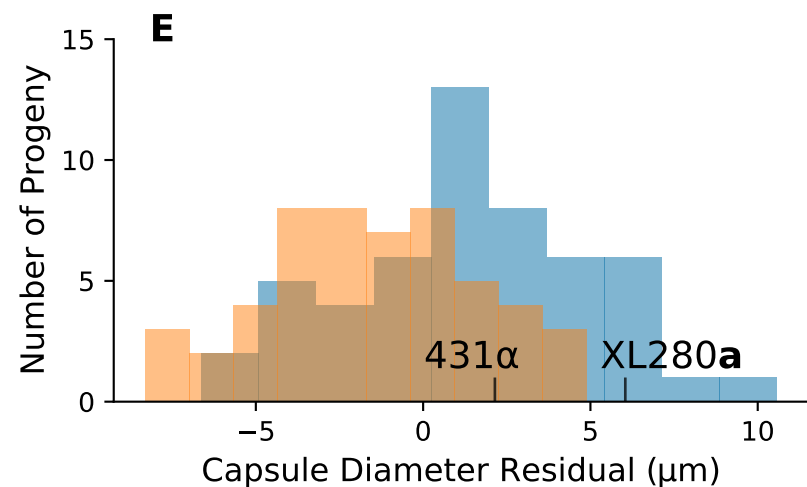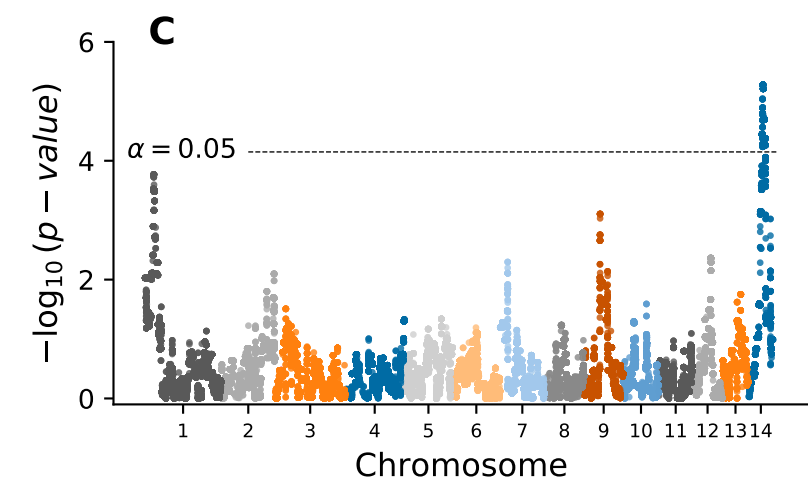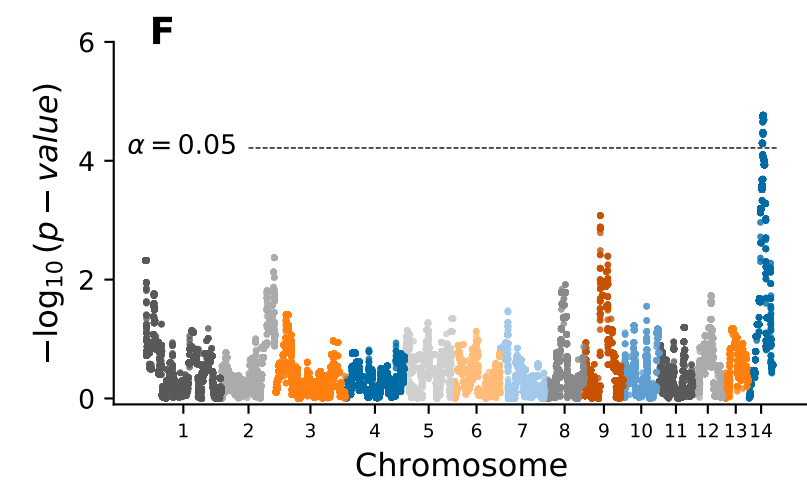

Supplement: S2 Fig — A—C) Analysis of variation in cell diameter (y-axis) as a function of cell and capsule diameter (x-axis, A), a histogram of the cell diameter residuals used in QTL mapping (B), and associated Manhattan plot (C). D—F) Analysis of variation in capsule diameter (y-axis) as a function of cell and capsule diameter (x-axis, D), a histogram of the capsule diameter residuals used in QTL mapping (E), and temporaassociated Manhattan plot (F). Grey lines and shaded regions in A and D represent regression models and associated 95% confidence intervals. The variation explained by these models is annotated within each plot. For both cell and capsule diameter residuals a QTL is detected on chromosome 14. Dotted horizontal lines represent significance thresholds from permutation tests. Progeny cell and capsule diameter and cell and capsule diameter residual values are colored by the chromosome 14 QTL allele. (PDF) [file pgen.1009313.s005.pdf]

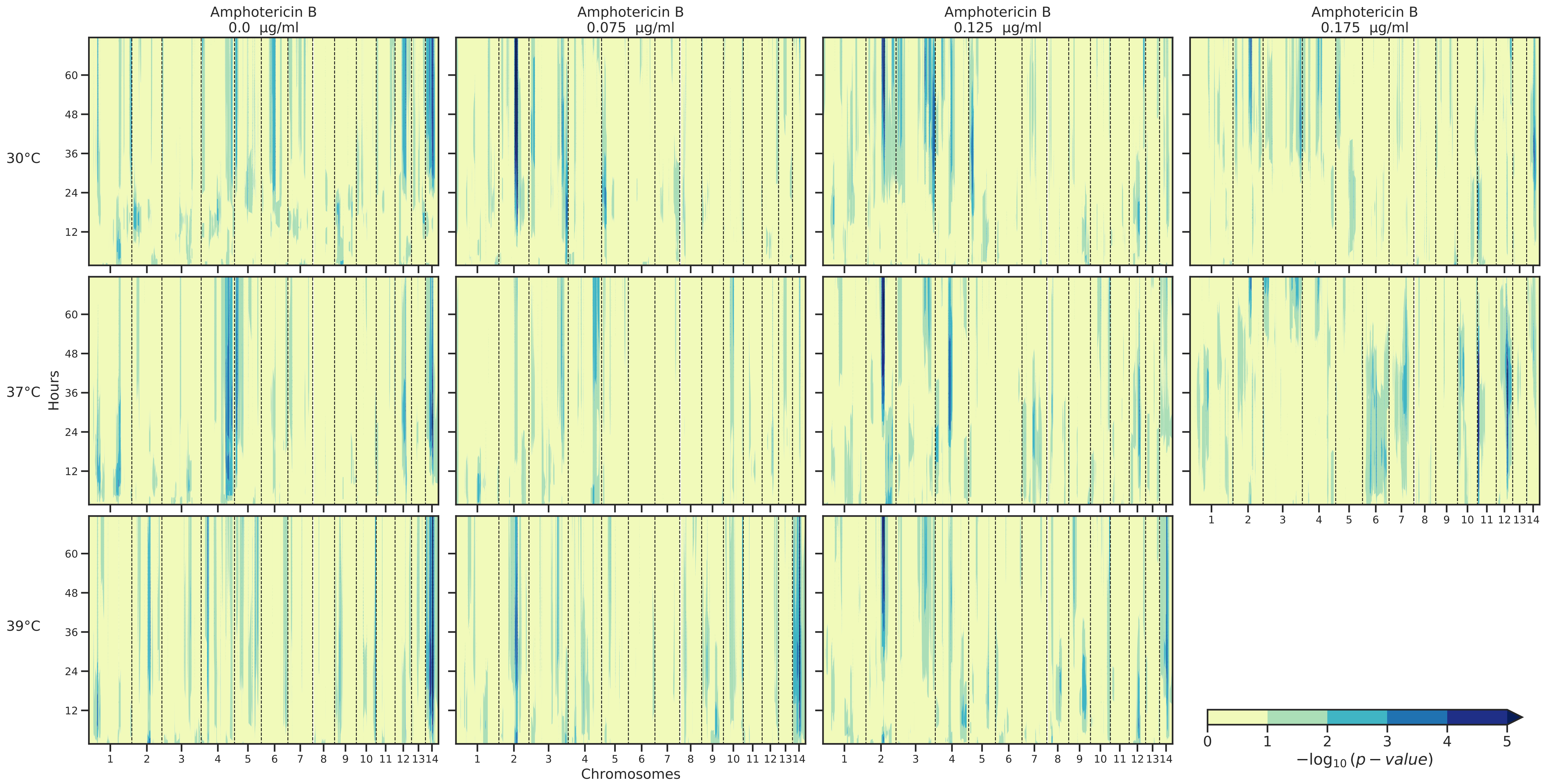

Supplement: S3 Fig — Genome-wide Manhattan heat maps of association between genotype and phenotype across 72 hour for combinations of temperature (rows) and amphotericin B (columns) concentrations in Fig 3. Across combinations of temperature and amphotericin B stress, the median growth AUC of segregants, calculated every 15 minutes for each 72-hour time course, was regressed onto the parental genotypes of XL280a and 431α. The yellow to blue colors depict the strength in association (as measured by the −log10(p−value) from the linear regression) between the growth AUC values and 3,108 bi-allelic haploblocks across segregants (x-axis) along the 72-hour time course (y-axis). (PDF) [file pgen.1009313.s006.pdf]

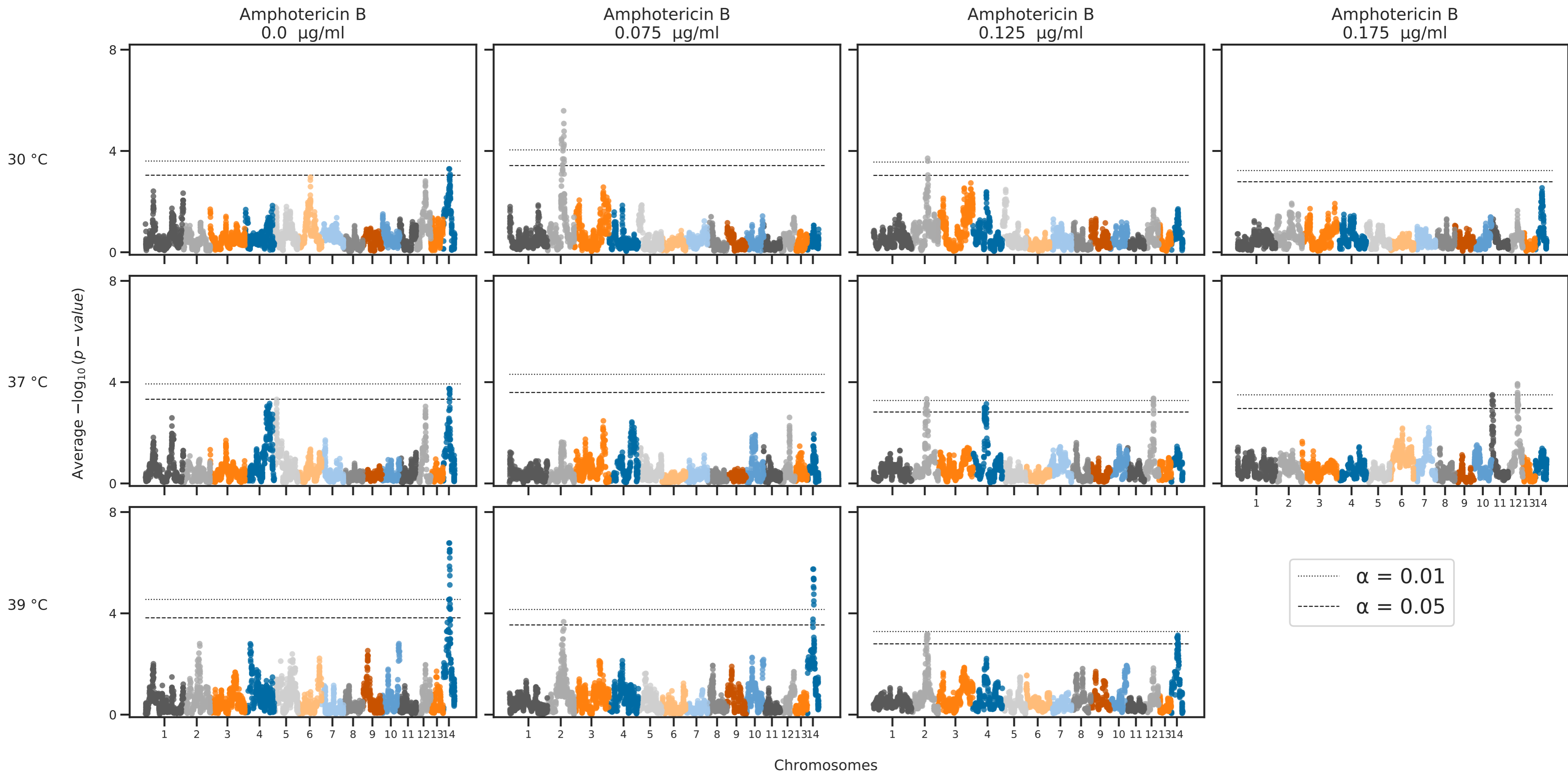

Supplement: S4 Fig — Genome-wide Manhattan plots of average association across time between genotype and phenotype for combinations of temperature (rows) and amphotericin B (columns) stress. For each experimental condition in Fig 3, the median growth AUC of segregants across the 72-hour time course was regressed onto the parental genotypes of XL280a and 431α. The x-axis represents positions along chromosomes (separated by colors) of 3,108 bi-allelic genetic variant sites, collapsed into haploblocks across segregants, and the y-axis is the average association across the 72-hour time course between genotype and the growth AUC values. Significance thresholds (horizontal dashed and dotted lines) were determined via permutation. (PDF) [file pgen.1009313.s007.pdf]

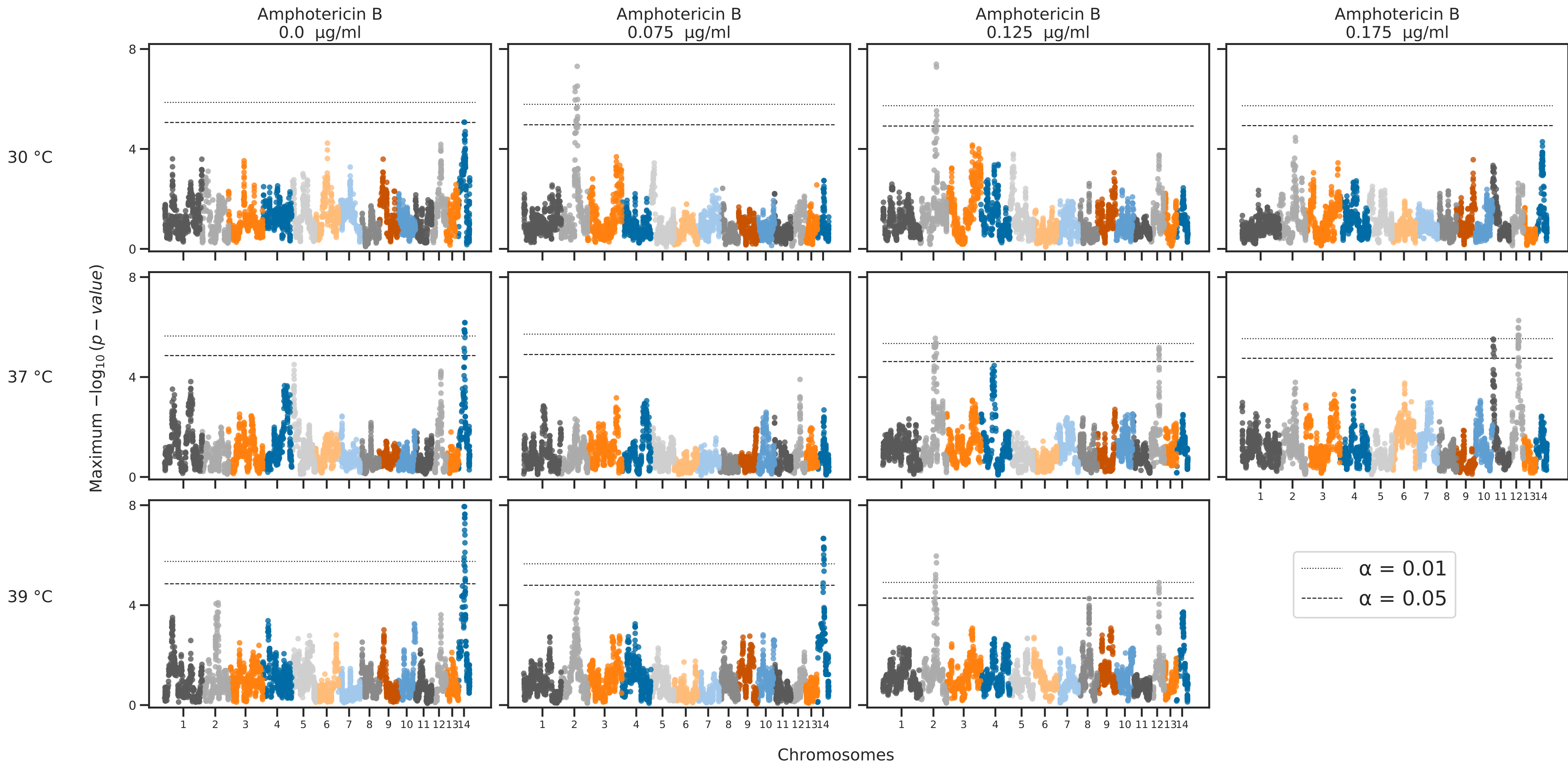

Supplement: S5 Fig — Genome-wide Manhattan plots of maximum association across time between genotype and phenotype for combinations of temperature (rows) and amphotericin B (columns) stress. For each experimental condition in Fig 3, the median growth AUC of segregants across the 72-hour time course was regressed onto the parental genotypes of XL280a and 431α. The x-axis represents positions along chromosomes (separated by colors) of 3,108, bi-allelic genetic variant sites, collapsed into haploblocks across segregants, and the y-axis is the maximum association across the 72-hour time course between genotype and the growth AUC values. Significance thresholds (horizontal dashed and dotted lines) were determined via permutation. (PDF) [file pgen.1009313.s008.pdf]

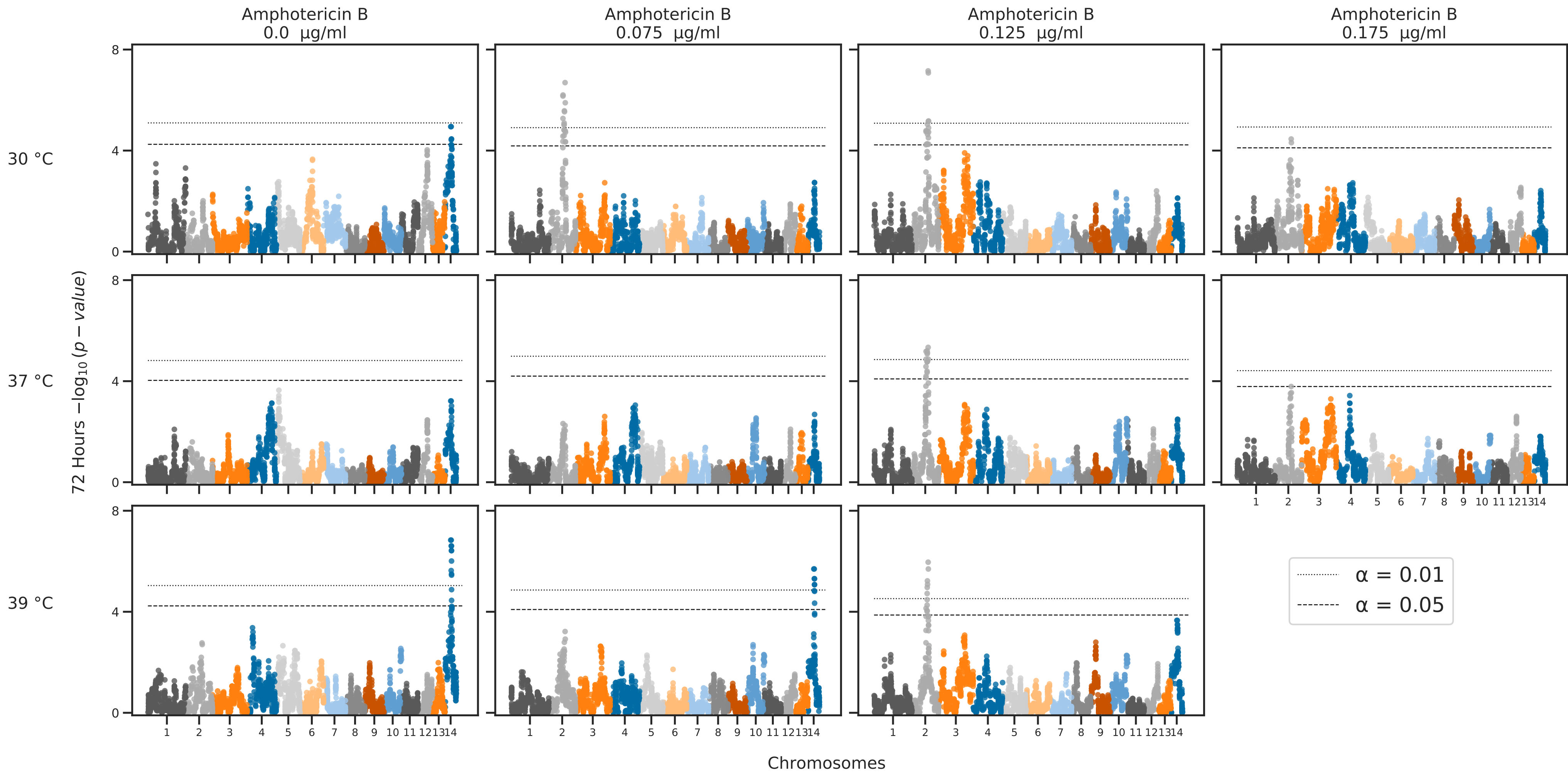

Supplement: S6 Fig — Genome-wide Manhattan plots of association between genotype and phenotype for combinations of temperature (rows) and amphotericin B (columns) stress. For each experimental condition in Fig 3, the median growth AUC of segregants at 72 hours of segregants was regressed onto the parental genotypes of XL280a and 431α. The x-axis represents positions along chromosomes (separated by colors) of 3,108 bi-allelic genetic variant sites, collapsed into haploblocks across segregants and the y-axis is the association between genotype and the growth AUC values at 72 hour. Significance thresholds (horizontal dashed and dotted lines) were determined via permutation. (PDF) [file pgen.1009313.s009.pdf]

**A**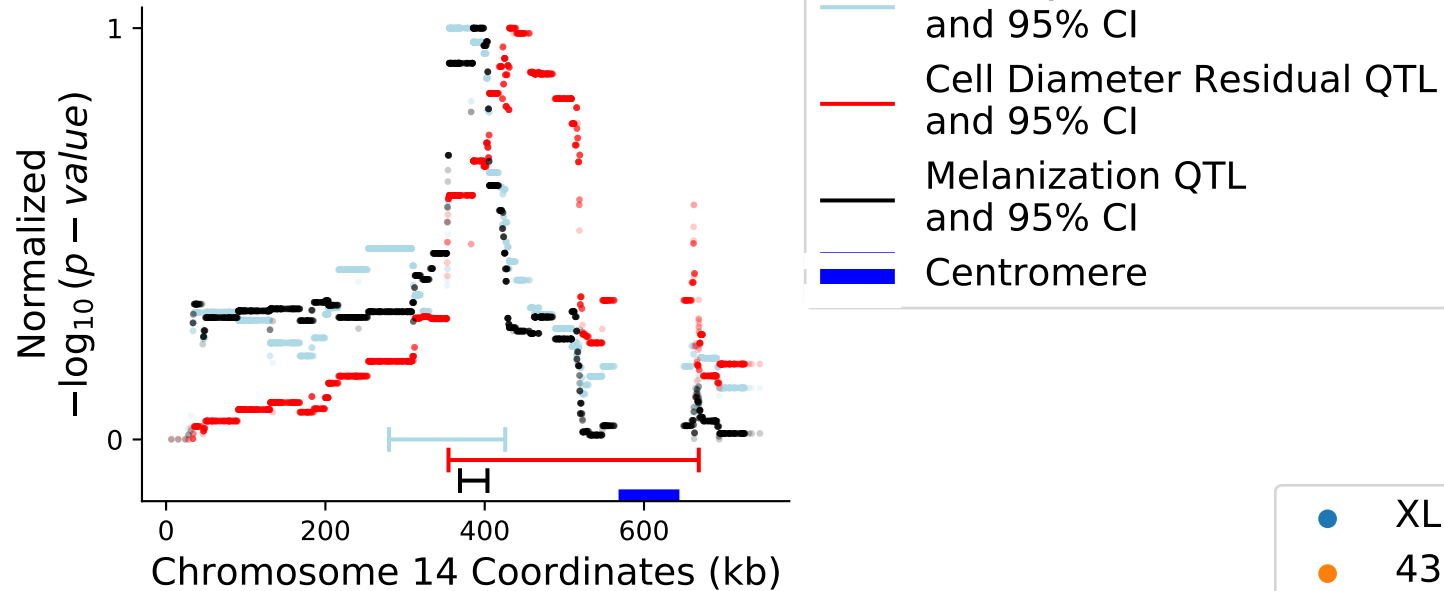**B**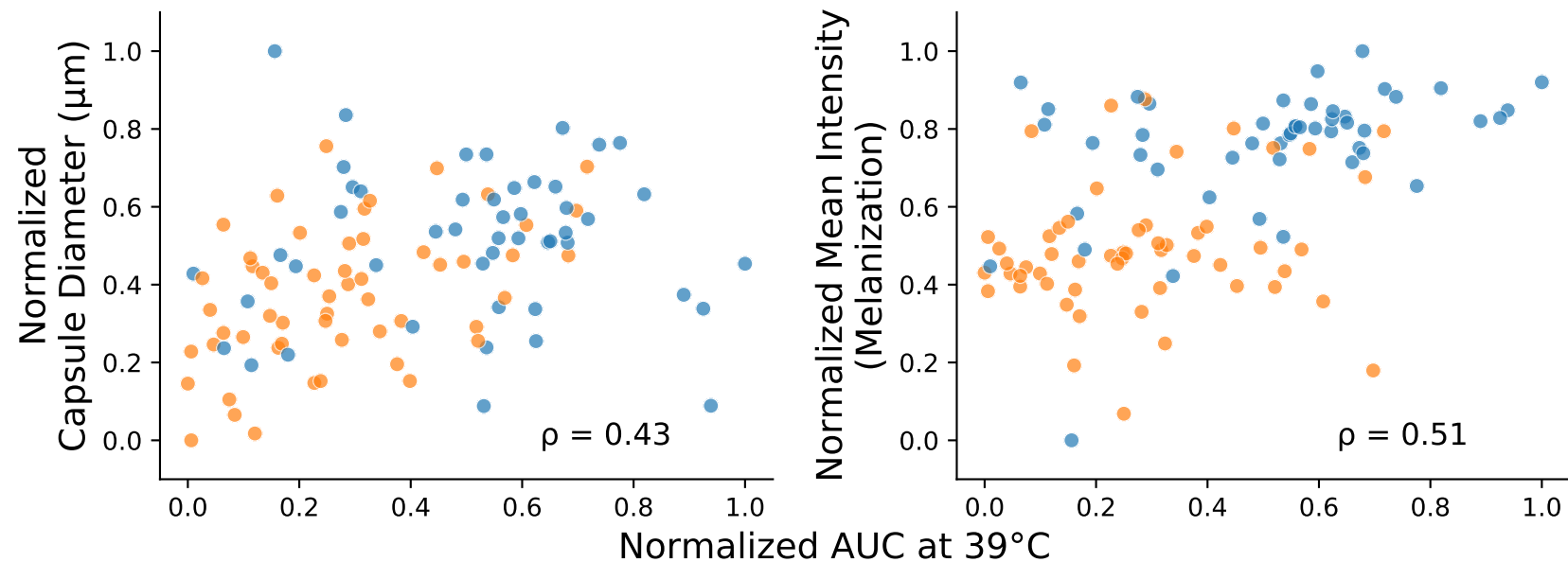

Supplement: S7 Fig — A) The three QTL and associated confidence intervals (CI) for area under the curve at 39°C (light blue), cell diameter residuals (red), and melanization (black). Horizontal bar bells represent 95% confidence intervals. The location of the centromere on chromosome 14 is marked by a horizontal blue bar. B) Phenotypic relationships between capsule diameter and melanization (y-axis of left and right panels, respectively) as a function of growth at 39°C (x-axis). Progeny values are colored by the allele at peak of the melanization QTL in A; blue for XL280a and orange for 431α. The Spearman rank correlation (ρ) between each pair of phenotypes is annotated within each plot. All QTL and phenotypic values in A and B (respectively) are re-scaled—in order to share the same scale—using max-min normalization. (PDF) [file pgen.1009313.s010.pdf]

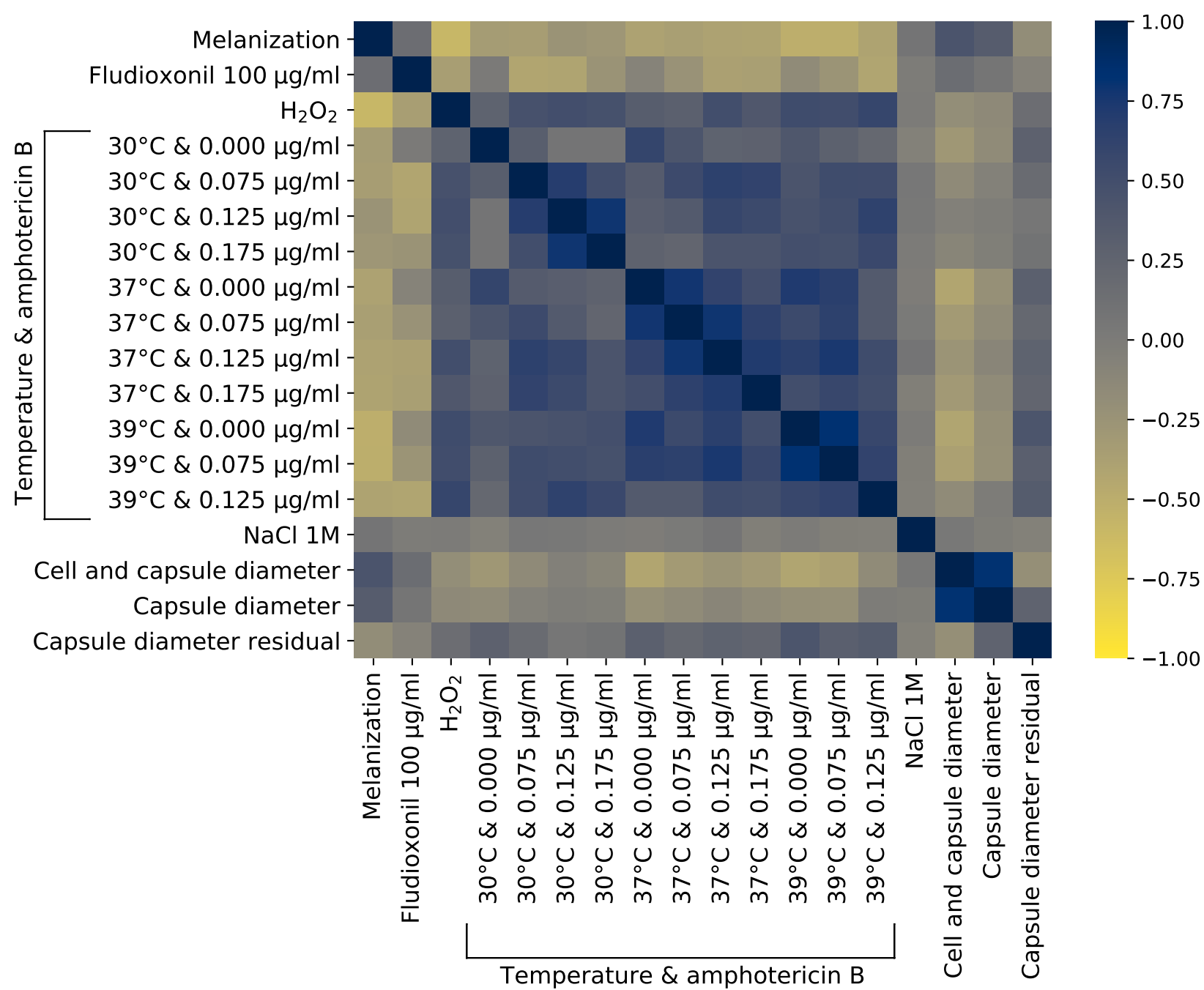

Supplement: S8 Fig — Spearman rank correlations between C. deneoformans phenotypes. (PDF) [file pgen.1009313.s011.pdf]

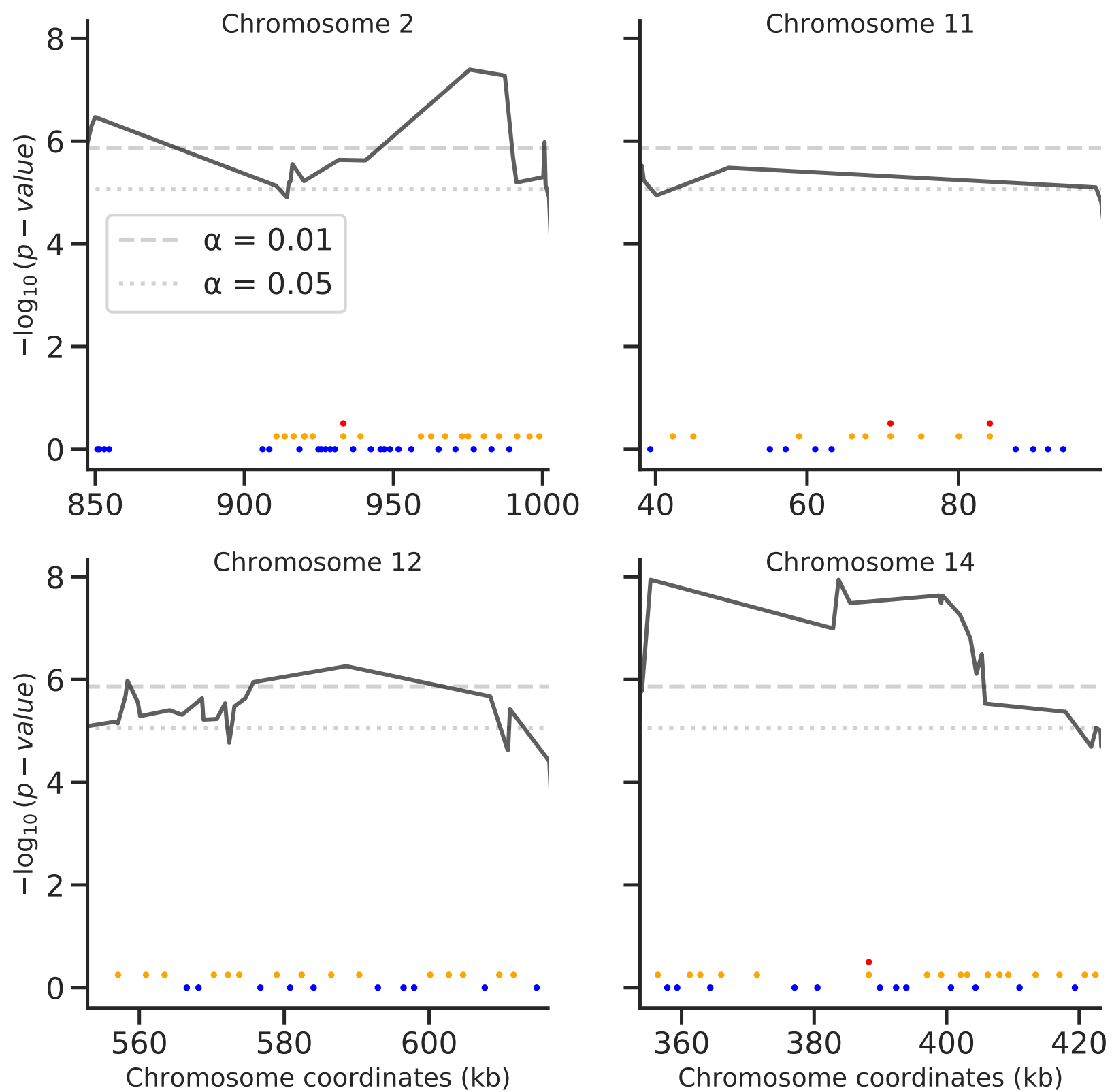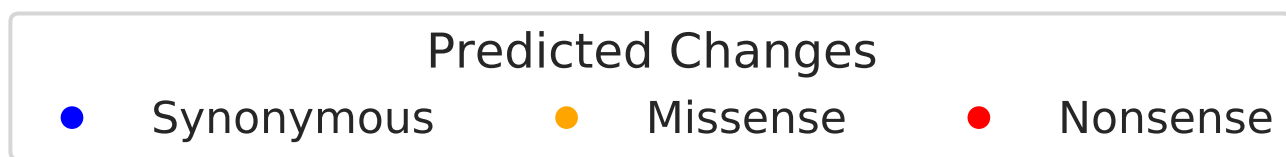

Supplement: S9 Fig — Locations of genes, relative to the four identified QTL (black curves). From the JEC21α reference genome [208], features were aligned to the XL280α reference [137] and the changes and differences in protein sequence between XL280a, XL280α, and 431α were predicted. Dots along the x-axis represent location of mapped genes, colors indicate predicted change between the XL280a (or XL280α) and 431α parental strains. (PDF) [file pgen.1009313.s012.pdf]

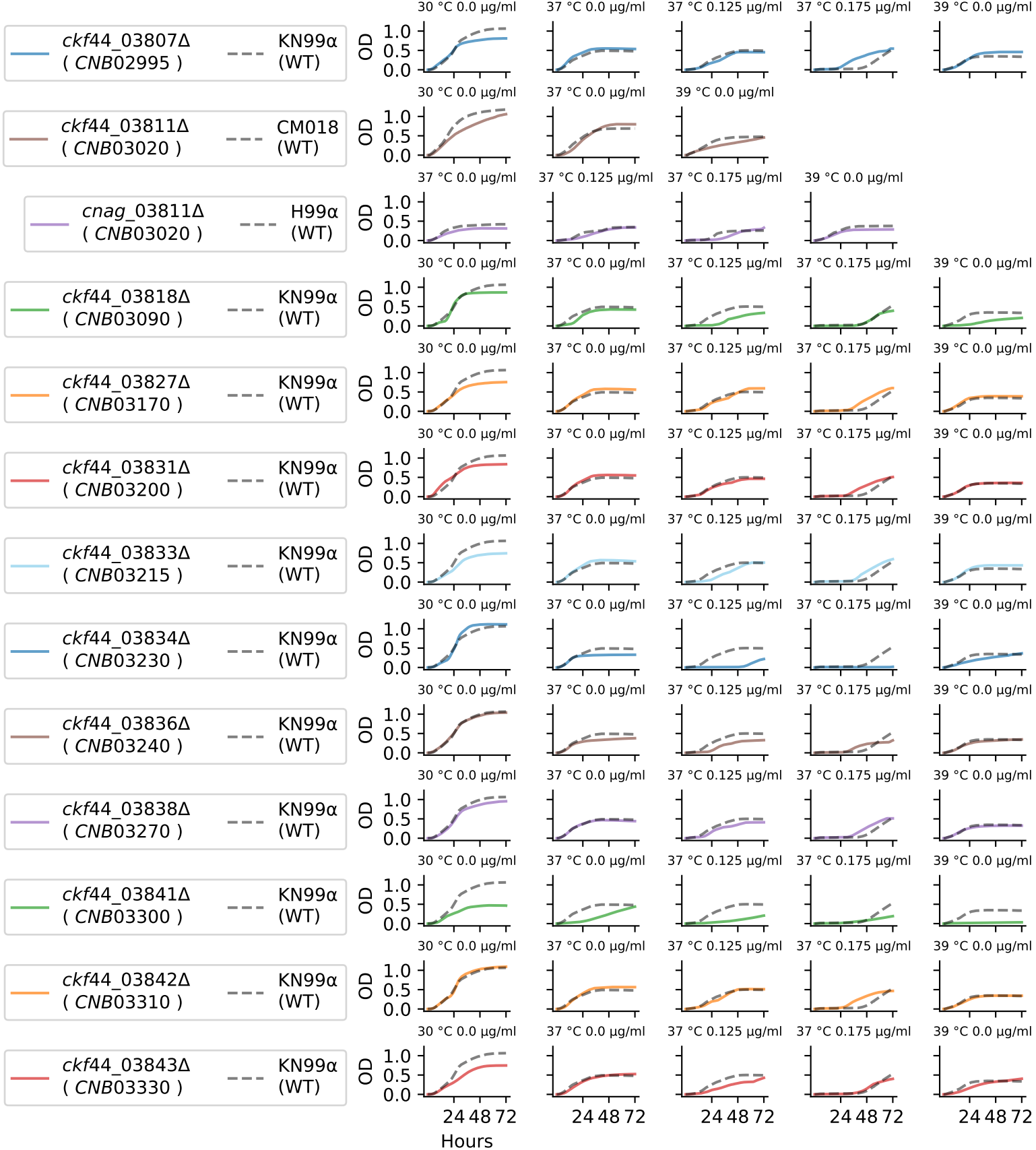

Supplement: S10 Fig — Growth of candidate deletion mutant strains for genes within the QTL along chromosome 2. The available deletion mutants (rows, solid curves) of genes within the QTL and the corresponding wild type, C. neoformans strain, were assayed for growth in liquid culture for 72 hour at high temperatures (30°, 37° and 39°C) and in the presence of amphotericin B (at 0.125 and 0.175 μg/ml). Legends on the far left show the gene names in the C. neoformans strain background with the corresponding C. deneoformans gene name. (PDF) [file pgen.1009313.s013.pdf]

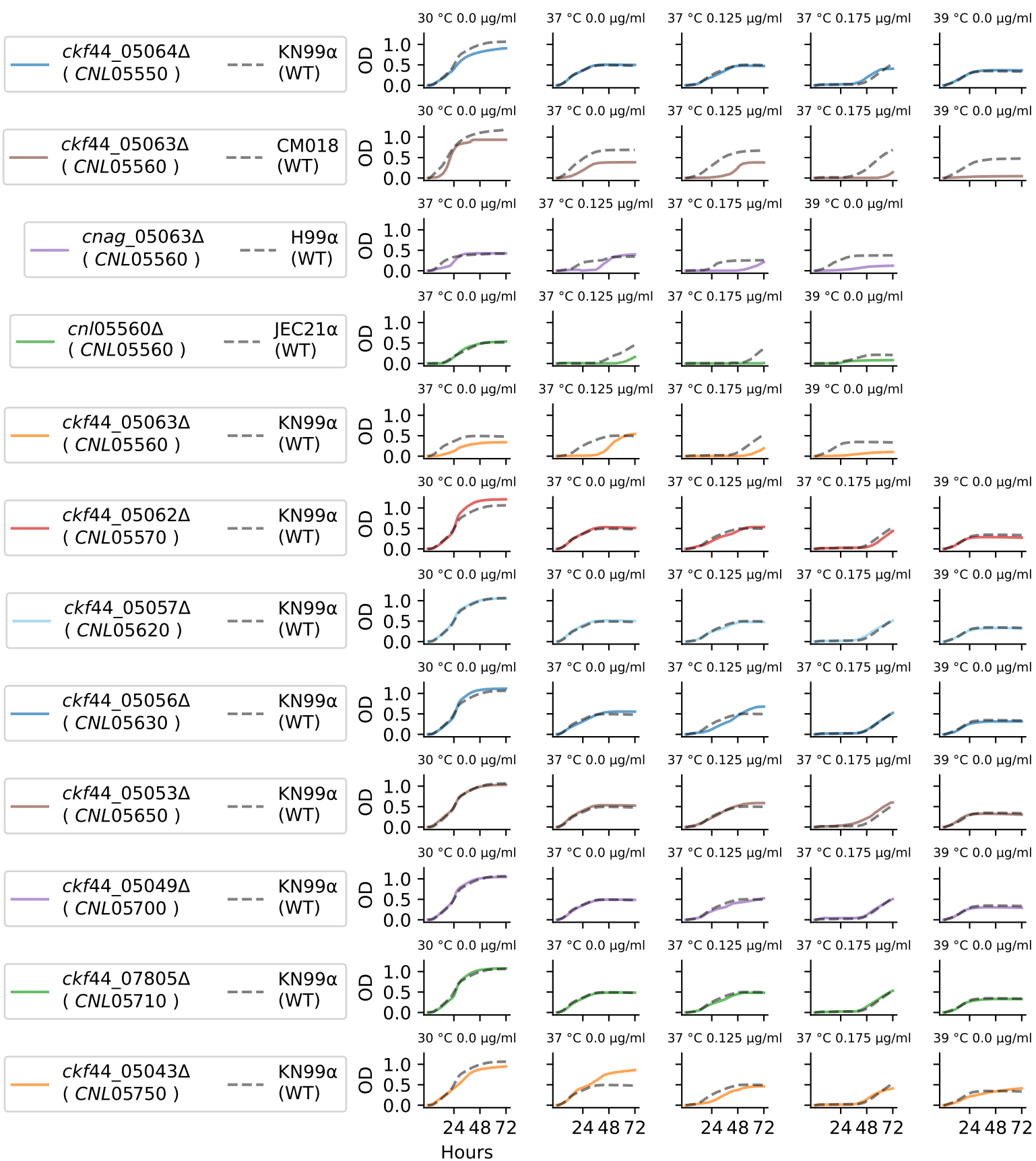

Supplement: S12 Fig — Growth of candidate deletion mutant strains for genes within the QTL along chromosome 12. The available deletion mutants (rows, solid curves) of genes within the QTL and the corresponding wild type, C. neoformans strain, were assayed for growth in liquid culture for 72 hour at high temperatures (30°, 37° and 39°C) and in the presence of amphotericin B (at 0.125 and 0.175 μg/ml). Legends on the far left show the gene names in the C. neoformans strain background with the corresponding C. deneoformans gene name. (PDF) [file pgen.1009313.s015.pdf]

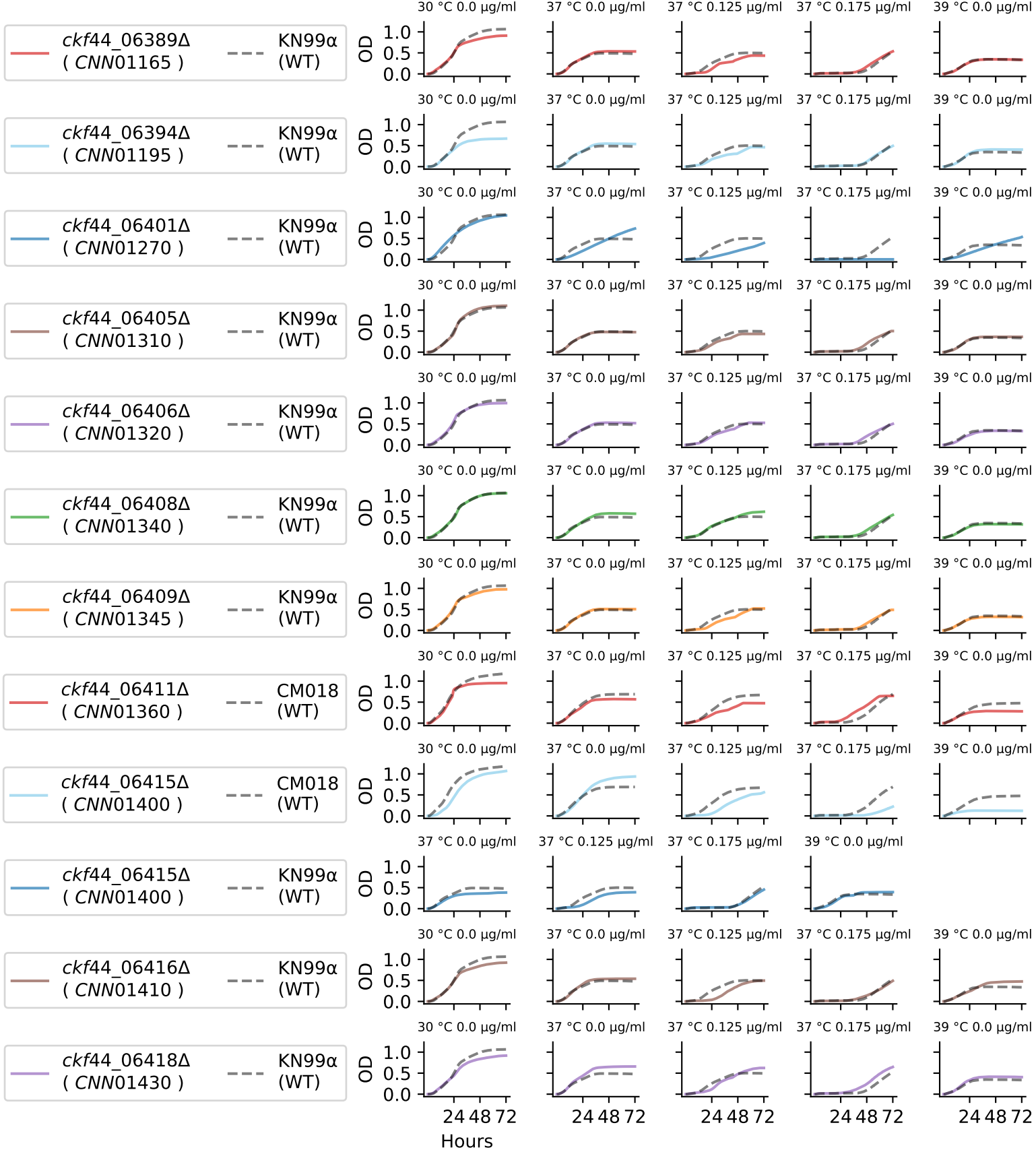

Supplement: S13 Fig — Growth of candidate deletion mutant strains for genes within the QTL along chromosome 14. The available deletion mutants (rows, solid curves) of genes within the QTL and the corresponding wild type, C. neoformans strain, were assayed for growth in liquid culture for 72 hour at high temperatures (30°, 37° and 39°C) and in the presence of amphotericin B (at 0.125 and 0.175 μg/ml). Legends on the far left show the gene names in the C. neoformans strain background with the corresponding C. deneoformans gene name. (PDF) [file pgen.1009313.s016.pdf]

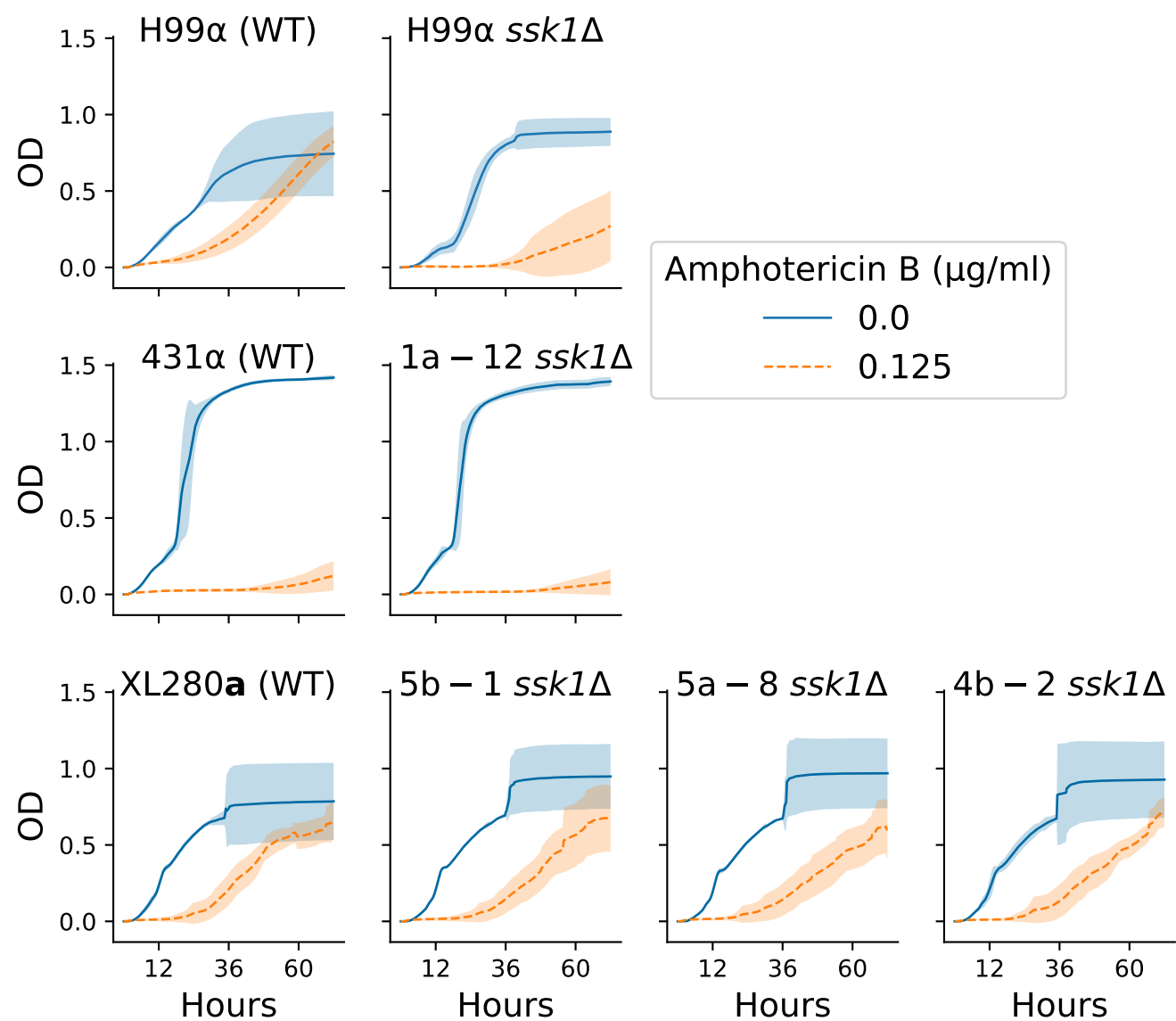

Supplement: S16 Fig — Growth of C. neoformans and C. deneoformans ssk1 deletion mutant strains. Across the rows and columns, the growth in liquid culture of wild type (WT) and ssk1Δ strains (y-axis, optical density, 595 nm) incubated for 72 hour (x-axis) at 30°C with and without amphotericin B (0.125 μg/ml). Rows separate strain backgrounds. Growth curves are shown in the first column for the C. neoformans WT strain, H99α (first row) and the C. deneoformans strains XL280a and 431α (last two rows, respectively). The growth curves of ssk1Δ strains, per background, are depicted in the second, third, and fourth columns. Solid blue curves and dashed orange curves represent mean growth curves with 0.0 and 0.125 μg/ml of amphotericin B (respectively) and shaded regions are 95% confidence intervals. (PDF) [file pgen.1009313.s019.pdf]

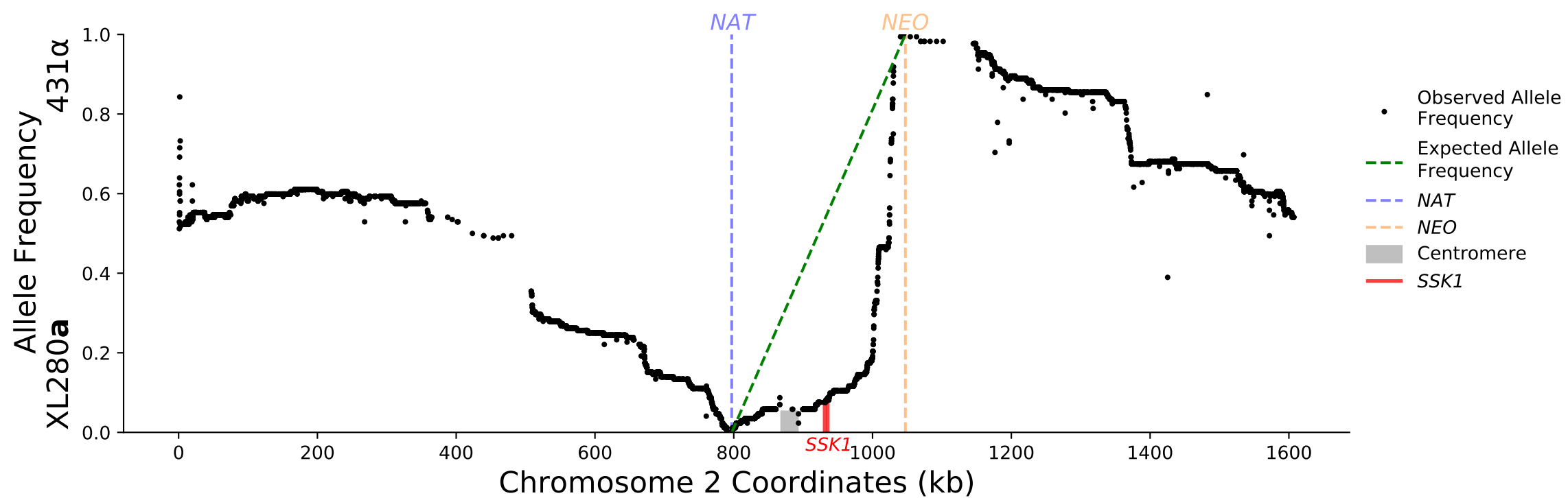

Supplement: S17 Fig — Allele frequency across bi-allelic, genetic variant sites of chromosome 2. Across chromosome 2 (x-axis) for the progeny generated from fine mapping, the position and allele frequency (y-axis) of genetic variants between the parental strains XL280a and 431α are shown. The position of the selectable markers transformed within the parental backgrounds are shown by vertical, blue and orange, dashed lines. The expected allele frequency in this region given the marker locations is shown with a green, dashed line. The positions of the centromere and the SSK1 gene are shown by grey and red rectangles, respectively. (PDF) [file pgen.1009313.s020.pdf]

**A**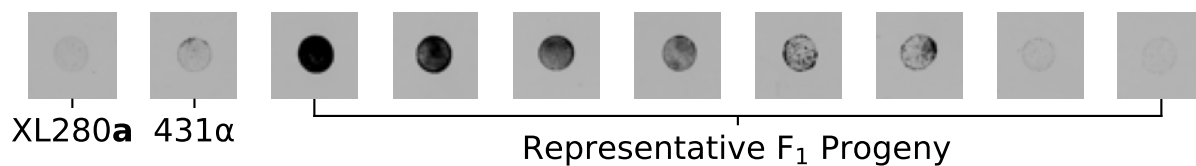**B**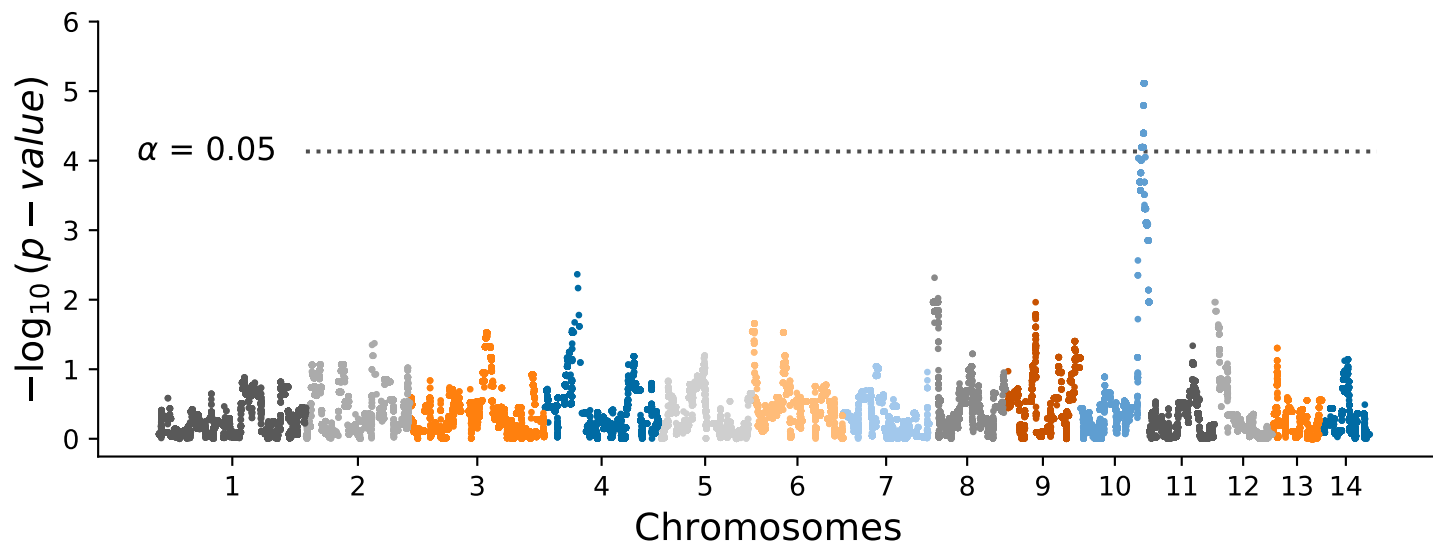**C**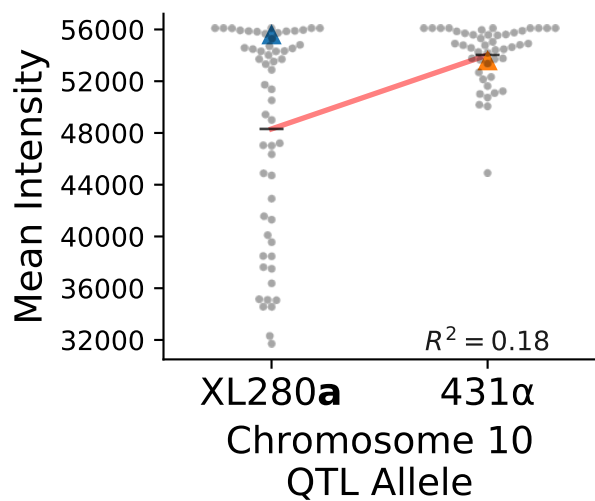

Supplement: S18 Fig — QTL analysis of variation in response to osmotic shock. A) Growth phenotypes of parental strains grown on media with 1M NaCl and range of phenoytpes of their segregants. B) Manhattan plot of the association between genotype and growth in response to osmotic shock. The x-axis represents chromosomal locations of haploblocks and the y-axis represents the strength in association between genotype and variation in growth as measured by the mean intensity from translucent scans. C) Mean intensity (arbitrary units) of segregants (gray dots) from translucent scans as a function of allele at the peak of chromosome 10 QTL. The parental phenotypes are displayed by blue and orange triangles. Black horizontal lines denote the phenotypic means by allele and a red line represents a regression model relating genotype to phenotype. The heritablity—estimated from this regression model—is ∼18 and annotated in black. (PDF) [file pgen.1009313.s021.pdf]

**A**

| <i>SSK2</i> | XL280a | 431α | Total |
|-------------|--------|------|-------|
| <i>RIC8</i> |        |      |       |
| XL280a      | 23     | 26   | 49    |
| 431α        | 42     | 13   | 55    |
| Total       | 65     | 39   | 104   |

**B**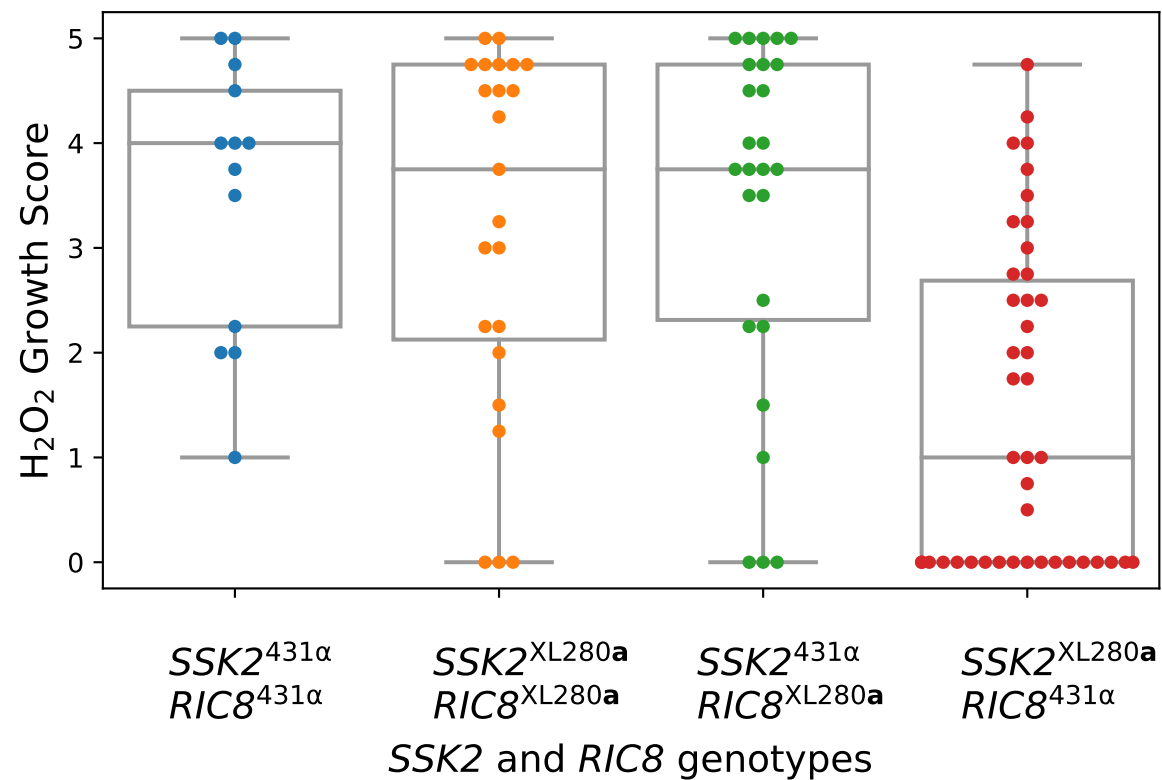**C**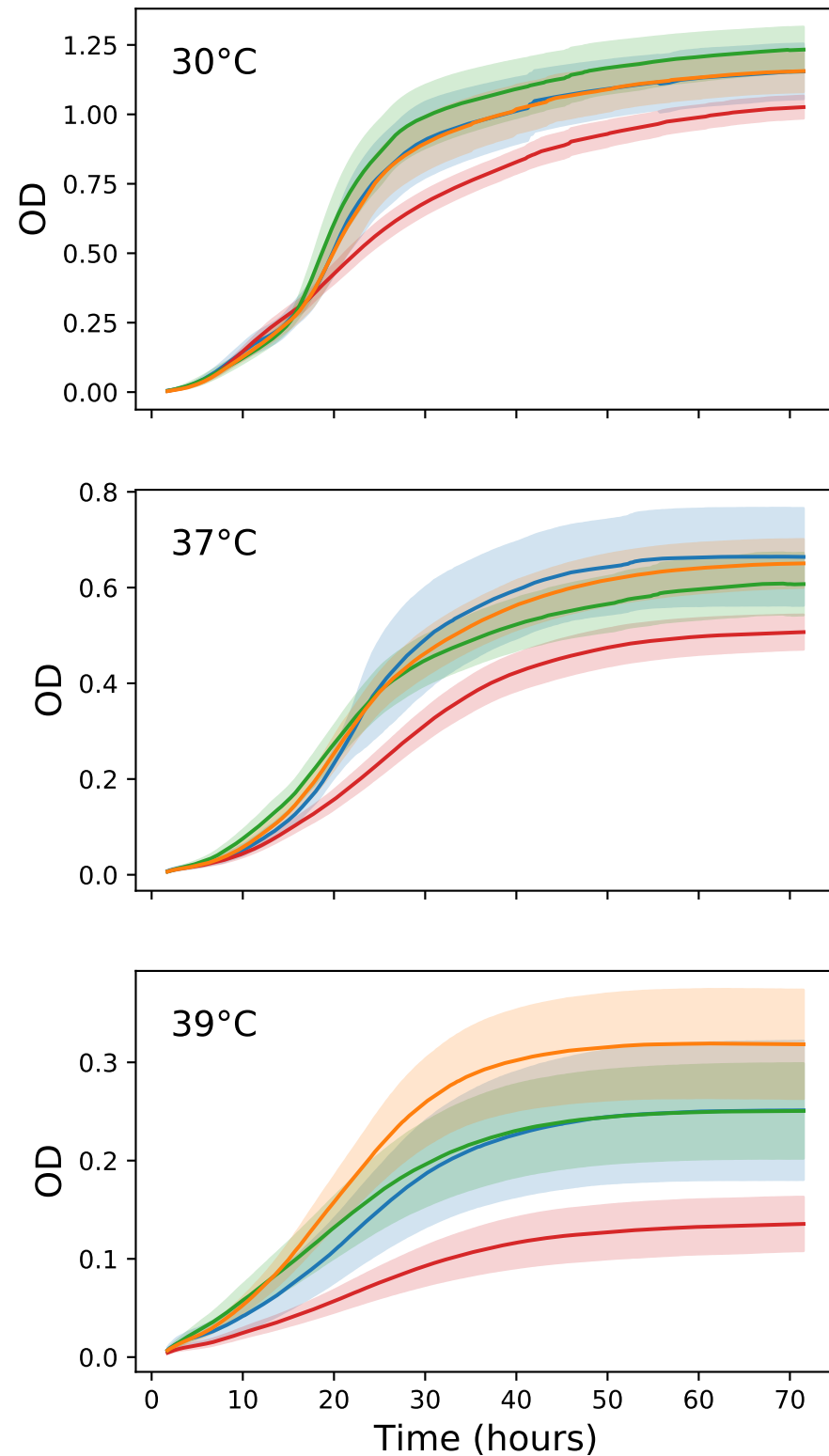

Supplement: S21 Fig — A) Contingency table of SSK2 (columns) and RIC8 (rows) alleles across segregants. B) Box- and swarm-plots of H2O2 growth scores (y-axis) by allelic combinations of SSK2 and RIC8 (x-axis). C) The mean growth curves (solid lines) and 95% pointwise confidence intervals (shaded regions) per allelic combination of SSK2 and RIC8 across temperatures. OD is optical density sampled at 595nm. In panels B and C, phenotypes are color coded by the combinations of SSK2 and RIC8 alleles listed in A. (PDF) [file pgen.1009313.s024.pdf]
