# Supplementary material for: Pleiotropy and epistasis within and between signaling pathways defines the genetic architecture of fungal virulence
Source: PLoS Genet. 2021 Jan 25;17(1):e1009313. doi: 10.1371/journal.pgen.1009313 (PMC7861560; doi:10.1371/journal.pgen.1009313)
Supplement: S11 Fig — Growth of candidate deletion mutant strains for genes within the QTL along chromosome 11. The available deletion mutants (rows, solid curves) of genes within the QTL and the corresponding wild type, C. neoformans strain, were assayed for growth in liquid culture for 72 hour at high temperatures (30°, 37° and 39°C) and in the presence of amphotericin B (at 0.125 and 0.175 μg/ml). Legends on the far left show the gene names in the C. neoformans strain background with the corresponding C. deneoformans gene name. (PDF) [file pgen.1009313.s014.pdf]

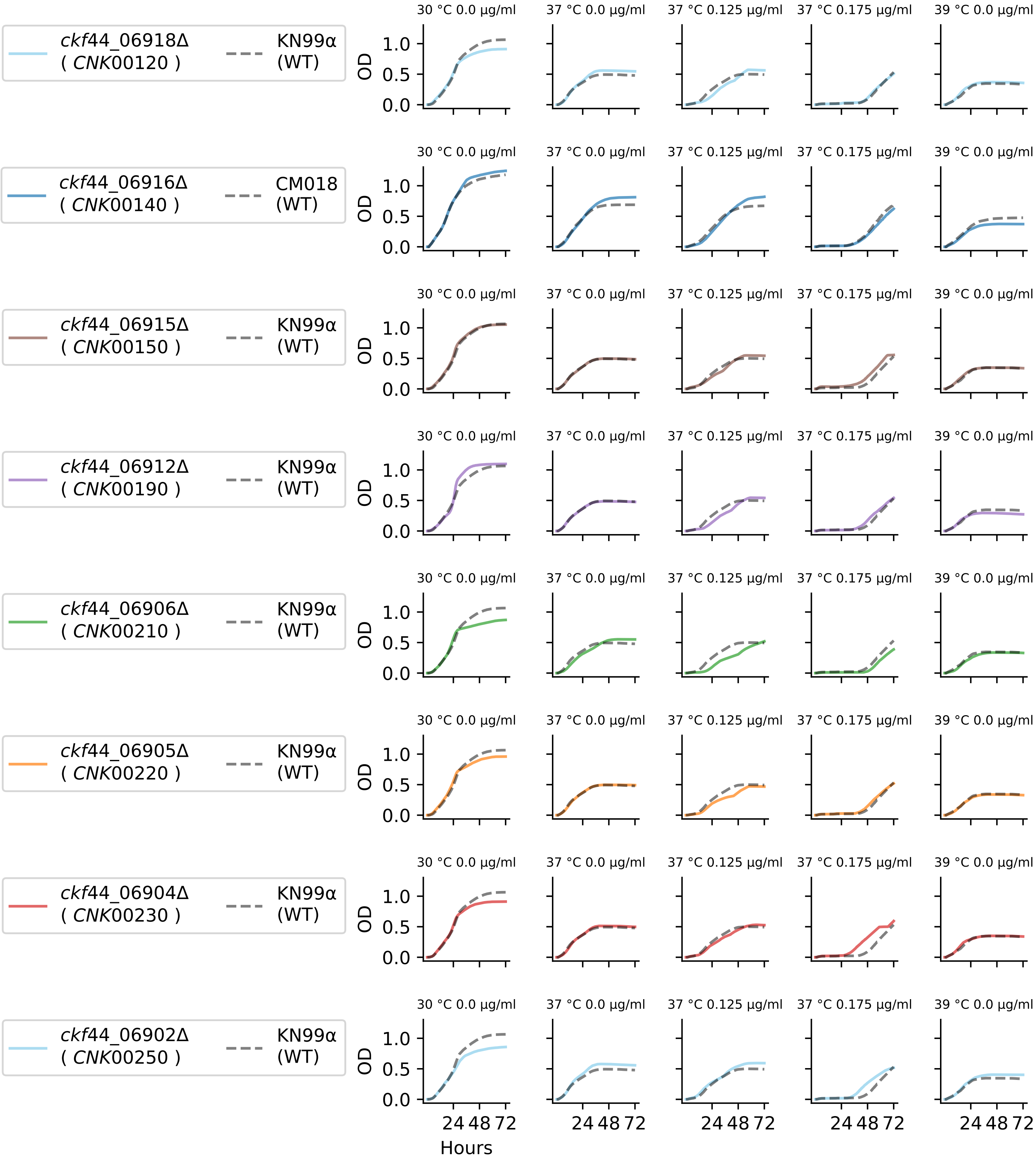

ckf44_06918Δ (CNK00120)
KN99α (WT)
ckf44_06916Δ (CNK00140)
CM018 (WT)
ckf44_06915Δ (CNK00150)
KN99α (WT)
ckf44_06912Δ (CNK00190)
KN99α (WT)
ckf44_06906Δ (CNK00210)
KN99α (WT)
ckf44_06905Δ (CNK00220)
KN99α (WT)
ckf44_06904Δ (CNK00230)
KN99α (WT)
ckf44_06902Δ (CNK00250)
KN99α (WT)
30 °C 0.0 µg/ml
37 °C 0.0 µg/ml
37 °C 0.125 µg/ml
37 °C 0.175 µg/ml
39 °C 0.0 µg/ml
OD
0.0
0.5
1.0
24 48 72
Hours
