# Supplementary material for: Pleiotropy and epistasis within and between signaling pathways defines the genetic architecture of fungal virulence
Source: PLoS Genet. 2021 Jan 25;17(1):e1009313. doi: 10.1371/journal.pgen.1009313 (PMC7861560; doi:10.1371/journal.pgen.1009313)
Supplement: S14 Fig — Exons are shown as large grey rectangles, while the introns, 5’ UTR, and 3’ UTR are shown as grey, horizontal lines. The positions of bi-allelic genetic variants between the parental strains, 431α and XL280a are marked by black, vertical lines. The positions of the predicted start and stop codons are annotated along the bottom of the gene bodies. Within the second exon of SSK1, an insertion site of a single nucleotide, present in the 431α parental strain is annotated and this insertion is predicted to cause a frame shift that leads to a downstream early stop-gain. Within the first, second, and third exons of SSK2, three SNPs are annotated that lead to non-synonymous changes. The allelic states of the last two non-synonymous changes in SSK2 have been previously identified by [143]. Within the third and last exon of RIC8, an in-frame codon deletion and shift in the predicted stop-codon (respectively) are seen in the 431α parental strain background. In the second to last exon of RIC8, a single-nucleotide polymorphism is present in the XL280a parental strain that is predicted to cause a premature stop. The local, predicted translations of the regions near these non-synonymous, genetic variants and associated amino acids are annotated in colored rectangles. (PDF) [file pgen.1009313.s017.pdf]

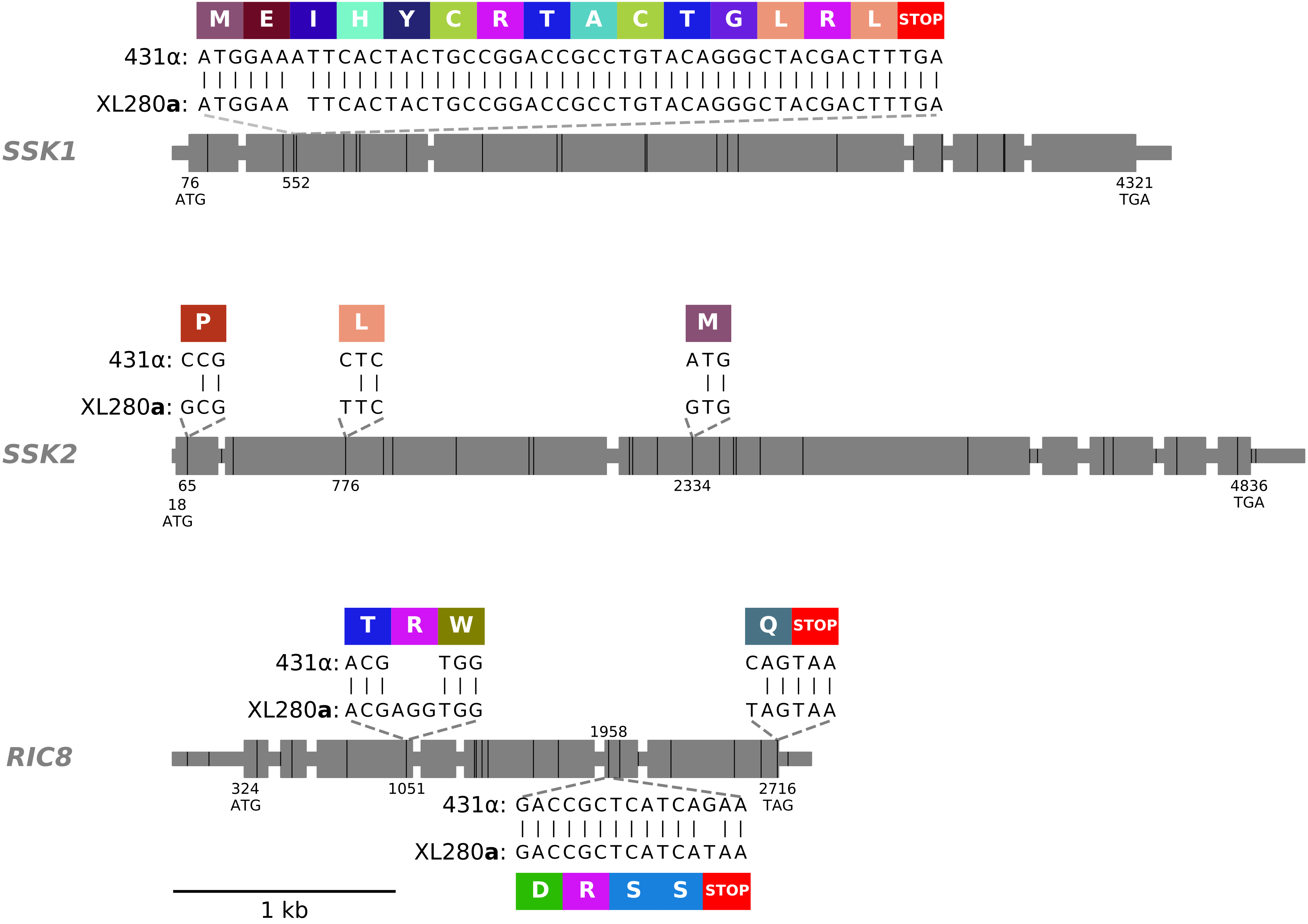

M E I H Y C R T A C T G L R L STOP
431α: ATGGAAATTCACTACTGCCGGACCGCCTGTACAGGGCTACGACTTTGA
XL280a: ATGGAA TTCACTACTGCCGGACCGCCTGTACAGGGCTACGACTTTGA
SSK1
76
ATG
552
4321
TGA
P
L
M
431α: CCG
CTC
ATG
XL280a: GCG
TTC
GTG
SSK2
65
18
ATG
776
2334
4836
TGA
T R W
Q STOP
431α: ACG TGG
CAGTAA
XL280a: ACGAGGTGG
TAGTAA
RIC8
1958
324
ATG
1051
2716
TAG
431α: GACCGCTCATCAGAA
XL280a: GACCGCTCATCATAA
D R S S STOP
1 kb
