# Supplementary material for: Pleiotropy and epistasis within and between signaling pathways defines the genetic architecture of fungal virulence
Source: PLoS Genet. 2021 Jan 25;17(1):e1009313. doi: 10.1371/journal.pgen.1009313 (PMC7861560; doi:10.1371/journal.pgen.1009313)
Supplement: S15 Fig — High temperature growth and melanization phenotypes of chromosome 14 candidate quantitative trait genes. The available deletion strains (rows) in the KN99α strain background of orthologous genes within the chromosome 14 QTL were assayed for high temperatures growth (37° and 39°C) in liquid culture and melanization on L-DOPA plates (columns, left to right respectively). Legends on the far left list the orthologous gene names in the C. deneoformans (JEC21α) and C. neoformans (H99α) background. Red and black curves display mean high temperature growth for the deletion strain and KN99α wild type (WT) strain (respectively) and shaded regions represent 95% confidence intervals. (PDF) [file pgen.1009313.s018.pdf]

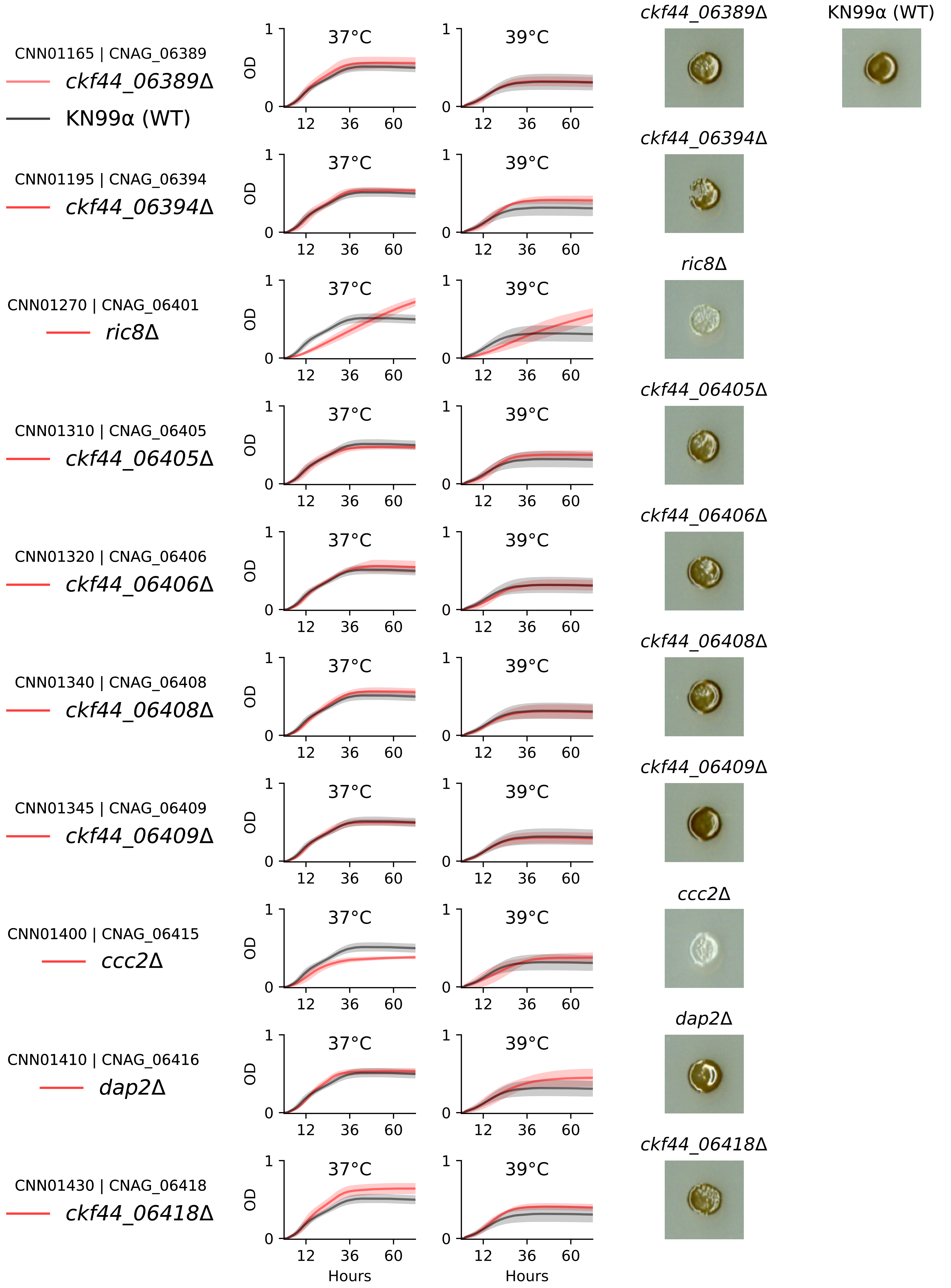

CNN01165 | CNAG_06389
ckf44_06389Δ
KN99α (WT)
CNN01195 | CNAG_06394
ckf44_06394Δ
CNN01270 | CNAG_06401
ric8Δ
CNN01310 | CNAG_06405
ckf44_06405Δ
CNN01320 | CNAG_06406
ckf44_06406Δ
CNN01340 | CNAG_06408
ckf44_06408Δ
CNN01345 | CNAG_06409
ckf44_06409Δ
CNN01400 | CNAG_06415
ccc2Δ
CNN01410 | CNAG_06416
dap2Δ
CNN01430 | CNAG_06418
ckf44_06418Δ
37°C
39°C
OD
Hours
12
36
60
0
1
