# Supplementary material for: Pleiotropy and epistasis within and between signaling pathways defines the genetic architecture of fungal virulence
Source: PLoS Genet. 2021 Jan 25;17(1):e1009313. doi: 10.1371/journal.pgen.1009313 (PMC7861560; doi:10.1371/journal.pgen.1009313)
Supplement: S19 Fig — The segregants represent the possible combinations of the SSK1 and SSK2 alleles from the XL280a strain, CF1730, and the 431α strains, CF1705, CF1706, CF1707. Within these progeny, of the 140 progeny with the SSK1 allele from XL280a parental strain and the SSK2 allele from the 431α parental strains (second to last row), 130 (93%) demonstrated sensitivity to fludioxonil (100 μg/ml). All other combinations of the parental alleles in the fine-mapping progeny demonstrated resistance to fludioxonil. (PDF) [file pgen.1009313.s022.pdf]

SSK1 Allele	*SSK2* Allele	# of Resistant	# of Sensitive
XL280**a**	XL280**a**	20	0
431α	XL280**a**	7	0
XL280**a**	431α	10	130
431α	431α	6	0
