# Supplementary material for: Pleiotropy and epistasis within and between signaling pathways defines the genetic architecture of fungal virulence
Source: PLoS Genet. 2021 Jan 25;17(1):e1009313. doi: 10.1371/journal.pgen.1009313 (PMC7861560; doi:10.1371/journal.pgen.1009313)
Supplement: S20 Fig — Chromosome 12 QTL and phenotypes for H2O2 and amphotericin B resistance. A) Normalized strength in association (y-axis) for the Chromosome 12 QTL for H2O2 (red) and amphotericin B resistance (black). The location of the candidate QTG, SSK2 is annotated. B) Median H2O2 growth score (y-axis) as a function of AUC at 37°C with 0.125 μg/ml of amphotericin B (x-axis). The Spearman rank correlation is annotated within the plot. Segregant values are colored by their peak allele at chromosome 12 and a histogram in the right panel counts the number of segregants (x-axis) per H2O2 score. (PDF) [file pgen.1009313.s023.pdf]

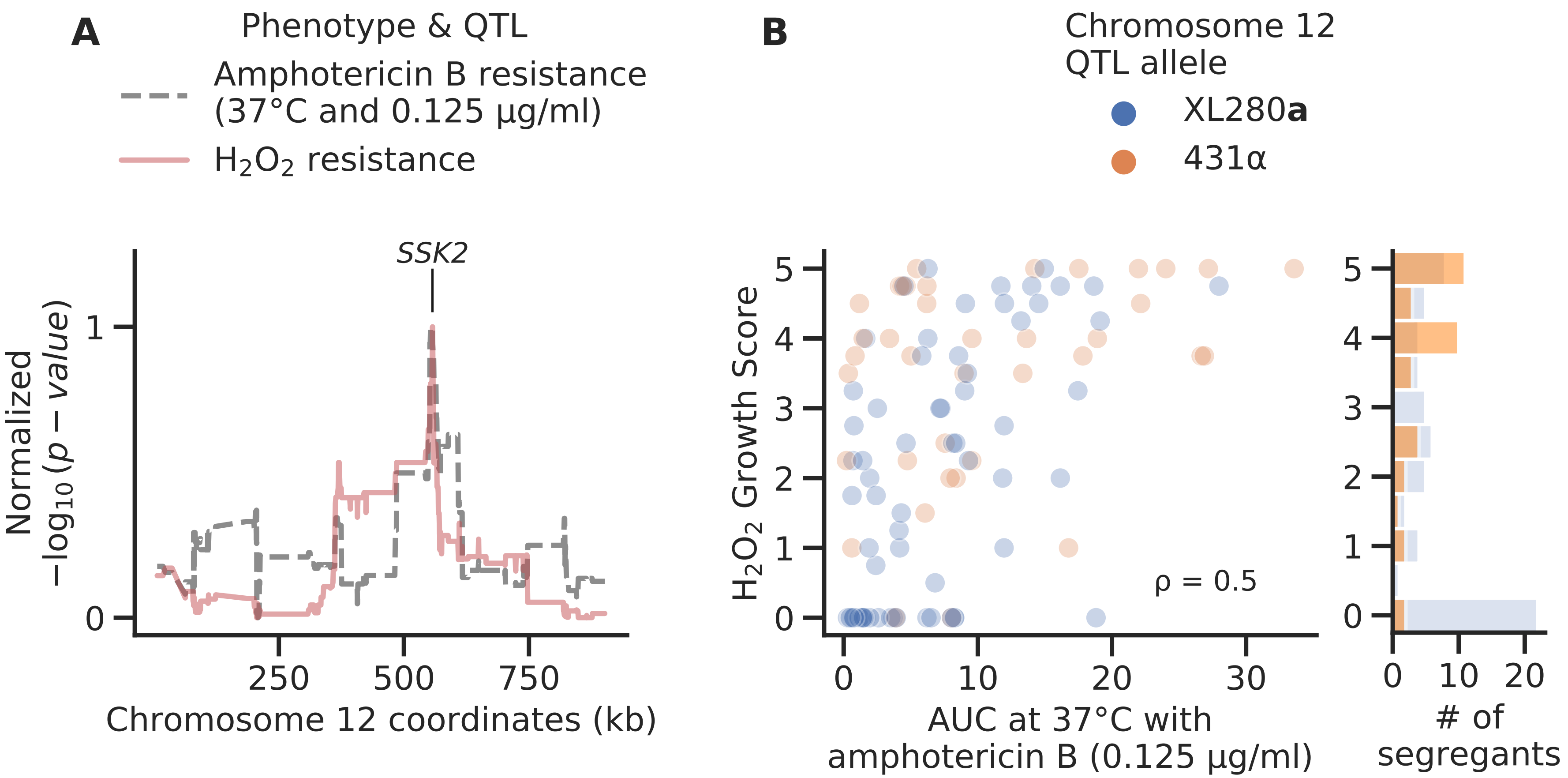
A
Phenotype & QTL
Amphotericin B resistance
(37°C and 0.125 μg/ml)
H_2O_2 resistance
SSK2
Normalized
$-\log_{10}(p-value)$
1
0
250
500
750
Chromosome 12 coordinates (kb)
B
Chromosome 12
QTL allele
XL280**a**
431α
H_2O_2 Growth Score
5
4
3
2
1
0
ρ = 0.5
0
10
20
30
AUC at 37°C with
amphotericin B (0.125 μg/ml)
0
10
20
of
segregants
